# The experience of men who participated in interventions to improve demand for and utilization of maternal and child health services in northern Nigeria: a qualitative comparative study

**DOI:** 10.1186/s12978-019-0761-2

**Published:** 2019-07-15

**Authors:** Olugbenga Oguntunde, Jabulani Nyenwa, Farouk Musa Yusuf, Dauda Sulaiman Dauda, Abdulsamad Salihu, Irit Sinai

**Affiliations:** 1 UKAid/ Nigeria MNCH2 Programme, No 17 Hospital Road, Nassarawa GRA, Kano State Nigeria; 2 Palladium, 20 Port Harcourt Crescent, Off Gimbiya Street, Garki, Abuja, Nigeria; 3 Palladium, 2nd Floor, Turnberry House, 100 Bunhill Row, London, EC1Y 8ND UK; 4 Society for Family Health, No 8 Port Harcourt Crescent, Area 11, Garki, Abuja, Nigeria; 5 Palladium, 1331 Pennsylvania Avenue NW, Suite 600, Washington, DC, 20004 USA

**Keywords:** Maternal and child health, Male involvement, Male support group, Perceptions, Danger signs, Northern Nigeria

## Abstract

**Background:**

Men in northern Nigeria are considered the leaders and ultimate decision makers, including decisions about health-related behaviours of their wives and children. Yet many men in the region consider pregnancy and childbirth to be in the woman’s domain (even if she cannot make related decisions), and may not see a need to educate themselves on the issues. These dynamics directly influence demand for, and utilization of, maternal, newborn, and child health services. This study examines an intervention that educated married men in northern Nigeria about health issues related to pregnancy, labour, delivery, and the postpartum period, as well as newborn and child health, through participation in male support groups. The curriculum also included interpersonal relationship and household decision making, with an emphasis on the need for men to give their wives standing approval to seek health services as needed, for themselves and their children.

**Methods:**

We conducted 12 focus group discussions with married men in Kaduna and Katsina states in northern Nigeria – half with men who had participated in the male support groups and half with men from areas that the intervention had not reached. Analysis was thematic, focusing on participants’ perceptions of the male support groups, the benefits of the intervention, and enablers and barriers to support group participation.

**Results:**

Perceptions of the male support groups were overwhelmingly positive. Participants internalized important messages they learned, which influenced their decisions related to the health of their wives and children. Some take it upon themselves to educate others in their communities about what they learned, and many say they see changes at the community level, with more utilization of maternal, newborn, and child health services.

**Conclusions:**

In the northern Nigeria context, educating men about danger signs of pregnancy, labour, delivery, newborn, and child health, is crucial to improving maternal and newborn health outcomes. Our intervention was successful not only in educating men, but also in converting some into advocates such that the effect of the intervention went beyond participants to the community. Programmes that aim to improve health-service utilization in northern Nigeria should consider scaling up this, or similar, interventions.

## Plain English summary

This study explored what married men thought about support groups that they participated in. The support group programme took place in northern Nigeria. It was designed to educate married men about health issues related to the health of their wives and their children. They learned, for example, about possible complications that can occur when the woman is pregnant, or when she delivers her baby, and what to do if a child is sick. We did the intervention in northern Nigeria, because health indicators in the region are among the poorest in the world. For this study we talked to groups of men who had taken part in the support groups and asked them what they thought of the support groups, how the support groups worked, why they joined the support groups, what they learned, and how they think the programme can be improved. We also talked to men who did not participate in the groups for comparison. Results show that the programme was successful. Participants reported better understanding of health issues of women and children. They also said that they see better health behaviours in the community, as a result of the intervention.

## Background

The patriarchal nature of societies in some parts of the world, particularly in many African countries, invests men with social and economic powers that enable them to exert significant control over their partners [1, 2]. In such settings, men manage their spouses’ fertility behaviours and their access to, and utilization of, available health-care services for themselves and their children [3, 4]. Men in these communities often see pregnancy and childbirth as exclusively in the woman problem, and yet women have no agency in this domain. Men still make all the decisions, but they often do not see a need to educate themselves on these issues [5]. This is the case in northern Nigeria, where men are considered the leaders and ultimate decision makers within the family, community, and society, and women believe that this is as it should be [6, 7]. Women are valued mainly for their reproductive functions and for the number of children they bear; yet their reproductive capacities are placed under strict control of men. Women are therefore highly dependent on their husbands, and defer to them in all household decisions, including decisions relating to their own and their children’s health [5, 8].

These gender dynamics and cultural norms significantly impact married women’s health-seeking behaviours, and directly influence their demand for, and utilization of, health services for themselves and their children [9]. Essentially, women cannot use these services without their husband’s explicit permission. Couple communication about these issues is poor, and men often do not give this permission, for reasons that range from not understanding the need, to fear of cost and mistrust of the health system [6, 10–12].

Several studies have described the important role of male involvement in utilization of maternal, newborn and child health, especially in restrictive societies, and the ultimate resulting improvements in health outcomes, such as increased contraceptive prevalence, improved birth preparedness, and increased utilization of maternal and child health services. Improved attitudes of women and men, when men have better understanding of women’s health needs, have also been reported [13–15]. It seems, then, that in the northern Nigerian context, involving men in women’s and children’s health has the potential to promote significant change and improve overall health outcomes for women and children.

In this context it is important to explore male interventions, designed to increase male involvement in women’s health. The 1994 International Conference on Population and Development recognized men as legitimate targets for sexual and reproductive health promotion. Since then, the literature abounds with examples of interventions and studies that do just that, usually with positive results [16, 17]. A number of studies in northern Nigeria found association between male knowledge of maternal and child health issues and healthy behaviours [18], but there were few interventions designed to improve men’s knowledge on these issues. A study in Kano, Katsina, and Zamfara states, all in North-West zone of the country, that trained 247 male motivators to counsel men, religious leaders, and traditional rulers in the communities on the importance and benefits of healthy timing and spacing of pregnancies and when to use modern contraceptives, resulted in increased contraceptive prevalence [19]. The 19% contraceptive acceptance rate in the project was significantly higher than the known figures (3%) for the region [20]. However, this result means that 81% of the counselled men did not receive a method for themselves or their wives, suggesting significant room for improvement.

### Nigeria maternal newborn and child health Programme

The Nigeria Maternal Newborn and Child Health Programme (MNCH2) is a five-year (2014–2019), UKAid-health funded programme, implemented in six northern Nigerian states. MNCH2 initiated an umbrella of demand-creation activities, designed to increase demand for, and utilization of, maternal, newborn, and child health services across the project’s states. Included is a men’s support group intervention, which is the focus of this study. The intervention created social groups for married men in their communities. Married men, with at least one wife aged 15–35, were invited to join the groups. Each group met 2–3 times. They were moderated by especially trained male group leaders from the same communities. Meeting schedule was flexible, and depended on the availability of participants. The group leaders had a minimum of primary school education and were literate. They were recruited through the health education and community mobilisation units of each local government area, and were trained by MNCH2 project staff to facilitate the men’s groups, as well as talk to men in the community in order to attract participants. Group activities were designed to improve men’s awareness of maternal and child health needs. The curriculum included discussion of reproductive health, family planning, danger signs in pregnancy, safe delivery, child health, immunization, health-related decision making, and basic communication skills. Key messages emphasised the need for men to approve their wives’ and children’s’ utilization of health services, and even provide their wives with a standing approval to seek health services when needed, if the husband is not present to provide immediate permission.

A total of 12,655 communities, in six northern Nigeria states, had been reached with the male support group intervention, by the time fieldwork started for this study. Since then the intervention continues to expand. This study explored the experience of men who participated in this intervention and the utility of the intervention in improving demand for, and utilization of, maternal, newborn and child health services.

## Methods

The study consisted of focus group discussions (FGDs) with married men in two northern Nigerian states where the MNCH2 programme is active: Kaduna and Katsina. FGD participants were married men who resided in intervention catchment areas, and who had participated in the men’s support groups; and others not involved in support groups, who resided in communities where the intervention had not been implemented.

The FGDs were designed to obtain a deep insight into men’s views about the intervention, including benefits and challenges, as well as their awareness of maternal, newborn, and child health issues, and their perceptions regarding their wives’ and children’s health. We conducted a total of 12 FGDs (6 in each state). Participants were identified from communities that were purposefully selected: half were communities where the MNCH2 programme had been active, and half where it had not. Communities were selected that were geographically close to each other in easy-to-reach areas. In each selected community, potential participants were approached and assessed by trained male recruiters for eligibility (married to a woman of aged 15–35; have at least one living child, and, in intervention areas only, men who had participated in a support group). Care was taken that no two participants in a focus group will have participated in the same male support group. Each FGD consisted of 8–12 participants, for a total of 54 participants in Kaduna and 58 in Katsina.

The FGD sessions were conducted in Hausa, the most commonly spoken language in the study areas. They were audio-recorded and transcribed into English. Analysis was an iterative process. All the transcripts were read several times and coded by more than one analyst. The coding process was inductive and undertaken in conjunction with the thematic question guides. The Atlast.ti software was used for analysis.

## Results

A total of 112 men, age 20–60 (mean 36.8) participated in 12 focus groups. Some 90% of participants were Muslim, and 91% were Hausa or Fulani, the two dominant ethnicities in the region, which are culturally similar [21]. All participants were married, and almost a third had more than one wife (maximum four). Mean number of living children was 4.7 (range 1–18).

We next report on participants’ responses to questions about how they learned of the support groups, why they joined, challenges to participation, and how the groups worked. Only participants who had participated in a support group are included in this part of the analysis. We then examine what participants know about danger signs in pregnancy, labour, and delivery, and their attitudes toward health care issues related to maternal and child health. Here we compare responses from participants who participated in support groups, and those who did not. We complete the analysis with perceptions of support group participants regarding benefits of the groups, and suggestions for improvements.

### Recruitment and participation

Support group participants became aware of the groups through different media. Some, for example, learned about them in health facilities or hospitals.

“*I heard of the mentoring programme when I took my wife to the hospital for antenatal care [ … ] at the hospital.” [Katsina, 32, intervention]*

Yet the work of the group leaders was evident. Some participants mentioned them by name, others described how the group leaders advertised the groups in the community.

“*[name] and [name] are those who introduced this programme to this community. They enlightened us about different health issues associated with pregnancy and most especially problems that require immediate attention.” [Katsina, 35, intervention]*

*“They came to the traditional ruler in my area, and they said they have attended a training [ … ] they also wanted the ruler to gather young men for them to inform them about the men support group.” [Kaduna, 45, intervention].*

Another strategy to advertise the support groups in the community was to hold some group meetings in the open. Since participants were not talking about their own personal issues there was no need for privacy. Holding group meetings in the open was a strategic decision, designed to attract men who would not know about the support groups otherwise.

When asked why they decided to join the support groups, FGD participants in all the groups said that they were told they would learn about health issues of women and children. It is also clear that the strategy of employing someone from the community to recruit participants worked, as men trusted someone they knew. Some joined the group because he was the one to suggest it.

“*I agreed to be mentored because of the relevance of the programme. It is a programme that talks about women and children’s health.”* [Katsina, 32, intervention].

*“I was motivated because the person that told us about the programme has indicated that he had benefited from what he learned from the programme, therefore he knows better ways to take care of his family health care than I am.”* [Kaduna, 30, intervention]

Responses made it clear that there was no standard way of conducting the groups. In some places they were regularly scheduled, others took place in times and places convenient to participants, and some were ad hoc. Support groups met in a variety of places, such as schools, places of worship, community buildings, and out in the open.

Participants in several FGDs emphasised that the instruction style of the group leaders was inclusive, free of discrimination or bias, and encouraging respondents to express their thoughts and emotions freely.

*“There is no discrimination as to whom the message should reach; everybody has equal chance of participation. There is no specified criterion other than interest of the prospective member.”* [Katsina, 38, intervention]

*“The mentors are good and polite. They know how to talk to people. Their attitude is what even gave us courage to remain in the programme.”* [Katsina, 38, intervention]

However, it seems that participation in the groups may be limited to men who are already more interested in, or better disposed toward the issues discussed, as participants talked about others who did not join the group because of ignorance. Participants who had taken part in support groups listed some challenges that made it difficult for them to participate fully. Most mentioned transportation difficulties in attending meetings – mostly distance and cost. The flexibility and variation in meeting times were both an enabler and a barrier. For some participants it gave the flexibility to join group meetings at times that were convenient to them. But other participants would have preferred fixed times that they could set their schedules around.

*The challenges I can remember [ … ]: (1) lack of financial support due to the nature of transportation to far places or fuel the motorcycles we have, (2) there is need for working materials like having the topics written on pamphlet [ … ], (3) there is also need for a fixed period or day, which may be treated as special day meant for this programme. [Kaduna 30, intervention]*

### Knowledge gained

The curriculum discussed in support group meetings include maternal, newborn, and child health issues and danger signs, as well as decision making and couple communication. Participant responses confirm that they indeed learned about these issues.

“*We discuss topics of child birth and nursing a baby or breast feeding a baby, and the health problems associated with newborn babies and children under 5 years, and the way to solve those problems or even avoid them.* [Kaduna, 27, intervention]

*“Maternal and child health problems, danger signs in pregnancy and in newborns, decision making and encouraging youth to donate blood to those who cannot afford it.”* [Katsina, 29, intervention]

Most participants who had taken part in the support groups felt that they had learned a lot from their participation. One specifically said that thanks to the programme, he can now identify different health problems relevant to his children and his wives, which he was not previously aware of.

*“The content of the programme is rich. It almost covers all aspects of maternal and child health and I appreciate it.”* [Katsina, 43, intervention].

In the course of the FGDs participants were asked to list danger signs in pregnancy, labour, and delivery, that may signal a need to seek urgent medical care. They were also asked about health problems to newborn babies. In Table 1, we list items that were spontaneously mentioned (without probing) in response to these questions, by FGD. Not all items spontaneously mentioned are listed in the table -- we only include items that were included in the curriculum. On the other hand, items that are taught are excluded if no one mentioned them spontaneously. These include, for example, lethargy in newborn babies.

**Table 1:** Knowledge items mentioned spontaneously

	Participated in support group						Did not participate in support group					
	Kaduna 1	Kaduna 2	Kaduna 3	Katsina 1	Katsina 2	Katsina 3	Kaduna 1	Kaduna 2	Kaduna 2	Katsina 1	Katsina 2	Katsina 3
Danger signs in pregnancy, labour and delivery												
Severe bleeding	X	X	X	X	X		X	X	X		X	
Severe headache	X	X	X	X	X		X			X	X	
High fever		X		X	X			X	X			
Blurred vision			X					X	X	X		
Swelling of the hand or face	X			X	X	X	X		X	X	X	X
Pelvic or abdominal pain	X	X	X							X	X	
Hypertension	X	X			X							
Anaemia or low blood pressure		X	X		X	X	X			X		
Baby does not move									X			
Water breaks without labour	X		X			X		X				
Convulsions		X		X								
Labour too long		X	X	X			X					
Placenta not delivered soon after baby		X					X					
Danger signs to newborn babies [first week]												
Difficulty of fast breathing	X		X	X				X		X	X	
Yellow skin or eye colour	X	X		X	X	X	X		X	X	X	
Poor sucking or feeding	X	X	X	X	X	X	X	X		X		X
Puss, bleeding, or discharge from around umbilical cord	X	X	X	X	X	X			X			
Baby very small	X	X		X	X	X		X	X		X	X
Convulsions or spasms		X	X									
Red or swollen eyes, or pus in eyes								X			X	
High fever	X	X	X	X			X	X	X			

While awareness of danger signs is far from comprehensive, it is clear that men who had attended support groups listed spontaneously more danger signs than men who did not. The total number of items mentioned ranged 7–15 in the FGDs of men who had participated in support groups (mean 11.3), and 3–9 for the FGDs of men in areas where the intervention was not implemented (mean 7.7). We asked a similar question also for children younger than 5 years. Instead of listing danger signs, participants mentioned known illnesses, such as typhoid, malaria, and measles, in both sets of FGDs. One participant said that it is easy to know, because children can talk and tell you what is wrong.

Participants in the FGDs for men who had attended support groups were unanimous that when they need health care for their families, they now go to a health facility. Some recognized that this is different than before the intervention.

*“Yes, we now know that it is important to take our pregnant women and newborn babies [to the nearest health centre] whenever we observe any health problem.”* [Kaduna, 36, intervention]

In contrast, in the FGDs for men who had not attended support group, some participants would go to health facilities, but others would prefer to get medical care from local patent medicine vendors. They said that the hospital is expensive (despite the fact that public health services in Nigeria are free of charge) and complained about quality of care.

*“The reason why some people go to the chemists [patent medicine vendors] is because they have shortage of money. So they will go there first before they would be able to afford the hospital, while if their sickness is healed they won’t go to the hospital again.”*[Katsina, 20, non-intervention]

*“There is just one problem, which happens mostly from the health care providers. For example, when you go to outpatient department [of health facilities] you can meet just 2 to 3 nurses attending to more than 100 people. Initially they will be enjoying the work but gradually it would reach a point they would get exhausted and start shouting inappropriately on patients and their relatives.”* [Katsina, 34, non-intervention].

Participants in all focus groups acknowledged that some men would not let their wives obtain health care. However, those who had participated in support groups also said that this is changing due to their group participation.

*“there are some men who do not like sending their wives to hospital due to ignorance or illiteracy. They view it as time wasting and resource consuming [ … ] and don’t give it any concern until when the conditions become critical.”* [Katsina, 39, intervention].

*“There are husbands that don’t permit their wives to go to the hospital because they are already used to the traditional medicines.”*[Katsina, 20, non-intervention].

*“In the past people only went to the hospital when they were seriously ill. Now women are allowed to go for antenatal care. For instance, my brother used to be very conservative but through my participation in this programme he changed his views and now sends his wife to hospital.”* [Katsina, 45, intervention]

### Benefits of male support groups

Participants who had taken part in support groups had only good things to say about them. Clearly, they benefited from group participation in their personal life. Not only did they gain knowledge, they also improved their interpersonal skills. It seems, also, that group participation gave them some prestige. One participant even said that he received promotion at work (from teacher to master of the school) as a result of his group participation.

*“Sincerely speaking, we have benefited a lot. We have received education which only people who attend medical school are privileged to have. On behalf of my people, we are grateful.”* [Katsina, 29, intervention]

*“My relationship with people around me and others I did not have contact with before have improved greatly, because people now greet me well wherever I go around. In an incident that occurred, a young girl collapsed, and her mother was confused. I told her what to do and subsequently donated blood to the young girl. I am now recognised by the young girl’s family and they offered me gifts on numerous occasions even after the incident.”* [Kaduna, 27, intervention]

Many participants also said that they had seen changes at the community level, with more people aware of the need to utilize health services, and more women and children receiving medical care. Some version of this was mentioned in all the focus groups.

*“the programme has enlightened both men and women in this community on the importance of going to the health facility, especially during pregnancy and delivery. In the past, a woman can stay in labour for almost three days and the common thing people will say is that she inherited it from one of her ancestors. But now we thank God, this programme has brought changes to this community on how we take care of women and children.”* [Katsina, 38, intervention]

“*The number of pregnant women attending antenatal care at the health facility has increased significantly because the facility is always busy with pregnant women daily. And the issue of child death [ … ] has reduced significantly due to proper caring from their mother, as a result of the information from this programme.”* [Kaduna, 38, intervention]

Moreover, it seems that the information participants receive through the programme resonates with some of them so strongly, that they take it upon themselves to educate others in the community.

*“On one occasion on a commercial bus, a man was telling a true story about a man that denied his wife permission to be taken to hospital and in the process the baby’s arm was broken before the woman could deliver. So, I tried to explain to them what I learned and some two men not from Kaduna said they are really happy to hear that information and that when they get home they will also enlighten on the issue*.” [Kaduna, 38, intervention]

### Suggestions for program improvement

While some support group participants were happy with the number of sessions, others said they would improve the program by adding more sessions to it. They also suggested that program continues in places where groups had already taken place. They said that since their participation they talked to others about what they learned, and those other men would benefit form support-group participation also.

One participant suggested that programme participants should have an organized visit to a health facility, to familiarize themselves with available services. Several others suggested a need to provide the group leaders with uniform or some means of identification. Another suggestion was to provide participants with written materials, such as pamphlets, containing the information they learned. Finally, several participants suggested that more men can be drawn to the programme if they were given a small allowance for their participation, to cover the cost of transportaion.

## Discussion

We presented the perceptions of men who participated in male support groups in Kaduna and Katsina states in northern Nigeria and found that they all had a very positive experience. We also included perceptions of men who had not participated in support groups, for comparison. More men are now informed, in intervention areas, on issues related to maternal and child health. They have a better grasp of danger signs to women and children that require immediate medical attention and are more open to health-service utilization. According to participants, the impact of the intervention goes well beyond the support-group participants themselves. Their wives and children benefit from their participation in their support groups, and they often take it upon themselves to also educate others in the community about what they learned.

Our findings confirm the importance of male involvement in maternal and child health, which is evident in the literature [12–14]. Given the patriarchal nature of northern Nigerian society, where men are expected to make all their household decisions, including those related to the health needs of their family [6, 7], the fact that participants feel so positively about the intervention, and have internalized some of the messages they learn so completely, is very encouraging.

Men’s involvement in women’s health has two broad objectives: (1) to improve women and children’s health outcomes, and (2) to promote gender equality [22, 23]. The intervention described in this paper can help promote the first, but not the second. One may even say, that our results suggest that men should be involved in maternal and child health so that they can make better decisions for women, rather than empowering women. We see this type of intervention, in the northern Nigeria setting, as a stepping stone. True, it does not promote women empowerment and gender equality. However, the literature shows that women are not yet ready to be empowered. They want their husbands to make health decisions for them [7]. An intervention such as this helps save lives, as cultural norms slowly and gradually change.

Of interest is the perception of respondents that some men do not let their wives and children receive care from health facilities because they perceive that this would be expensive. Maternal, newborn and child health services in most public healthcare facilities in northern Nigeria are free. However, while patients are not expected to pay for care and medications, they often have to pay for consumables (such as sterile gloves). Apparently, this cost is prohibitive to some.

Our findings suggest that the intervention’s strategy to have men from the community trained to become the group leader was successful, in that it increased recruitment to the groups, and made participants more comfortable with participating. However, recruitment could be improved further if the group leaders would be provided with a means to identify them as volunteers for the programme, and if they had printed materials to share with participants.

One concern is that participation may be somewhat limited to men who are already more interested in the issues discussed, as they are more open to participation after they are told that they would learn about maternal and child health. If this is the case, then the men who would benefit from such a program most are not included. Interventions must think creatively of ways to advertise the program, and the messages, more broadly. On the other hand, if participants are correct, and the effect of the intervention goes well beyond the participants themselves, then continued implementation of this program in the same communities may result in overall changes of perceptions at the community level over time.

Support group participants already perceive changes at the community, such as significant increase in health service utilization by women and children. However, if indeed there is such an increase, it may be attributed not only to the male support groups, but also to other demand-creation activities that were concurrently undertaken by the MNCH2 programmes in the same catchment areas. The umbrella of interventions included emergency transport schemes, safe space groups for married adolescents, and work with religious leaders [24].

## Conclusions

Given our findings, there is no question that the male support-group intervention is beneficial to men, their families, and to some extent their communities. The way that men were educated in our intervention was clearly effective, as they retained much of what they learned. Programmes that seek to improve demand for and utilization of MNCH services must think creatively about how to embed male support group interventions that provide effective mentoring and enlightenment while offering it at scale. If such programmes were scaled up in the region, demand for and utilization of health services is likely to increase, which should lead to improvements in health outcomes.

## Data Availability

Data sharing is not applicable to this article, because sharing qualitative focus group data may cause risk to participants’ confidentiality, and because participants were assured that the data they provide will not be shared.

## Abbreviation

FGD: Focus Group Discussion
